# Symptoms and patient factors associated with diagnostic intervals for pancreatic cancer (SYMPTOM pancreatic study): a prospective cohort study

**DOI:** 10.1016/S2468-1253(16)30079-6

**Published:** 2016-10-04

**Authors:** Fiona M Walter, Katie Mills, Silvia C Mendonça, Gary A Abel, Bristi Basu, Nick Carroll, Sue Ballard, John Lancaster, William Hamilton, Greg P Rubin, Jon D Emery

**Affiliations:** a University of Cambridge, Cambridge, UK; b University of Exeter, Exeter, UK; c Cambridge University Hospitals NHS Foundation Trust, Cambridge, UK; d Durham University, Durham, UK; e University of Melbourne, Parkville, VIC, Australia

## Abstract

**Background:**

Pancreatic cancer is the tenth most common cancer in the UK; however, outcomes are poor, in part due to late diagnosis. We aimed to identify symptoms and other clinical and sociodemographic factors associated with pancreatic cancer diagnosis and diagnostic intervals.

**Methods:**

We did this prospective cohort study at seven hospitals in two regions in England. We recruited participants aged 40 years or older who were referred for suspicion of pancreatic cancer. Data were collected by use of a patient questionnaire and primary care and hospital records. Descriptive and regression analyses were done to examine associations between symptoms and patient factors with the total diagnostic interval (time from onset of the first symptom to the date of diagnosis), comprising patient interval (time from first symptom to first presentation) and health system interval (time from first presentation to diagnosis).

**Findings:**

We recruited 391 participants between Jan 1, 2011, and Dec 31, 2014 (24% response rate). 119 (30%) participants were diagnosed with pancreatic cancer (41 [34%] had metastatic disease), 47 (12%) with other cancers, and 225 (58%) with no cancer. 212 (54%) patients had multiple first symptoms whereas 161 (41%) patients had a solitary first symptom. In this referred population, no initial symptoms were reported more frequently by patients with cancer than by those with no cancer. Several subsequent symptoms predicted pancreatic cancer: jaundice (51 [49%] of 105 patients with pancreatic cancer *vs* 25 [12%] of 211 patients with no cancer; p<0·0001), fatigue (48/95 [51%] *vs* 40/155 [26%]; p=0·0001), change in bowel habit (36/87 [41%] *vs* 28/175 [16%]; p<0·0001), weight loss (55/100 [55%] *vs* 41/184 [22%]; p<0·0001), and decreased appetite (41/86 [48%] *vs* 41/156 [26%]; p=0·0011). There was no difference in any interval between patients with pancreatic cancer and those with no cancer (total diagnostic interval: median 117 days [IQR 57–234] *vs* 131 days [IQR 66–284]; p=0·32; patient interval 18 days [0–37] *vs* 15 days [1–62]; p=0·22; health system interval 76 days [28–161] *vs* 79 days [30–156]; p=0·68). Total diagnostic intervals were shorter when jaundice (hazard ratio [HR] 1·38, 95% CI 1·07–1·78; p=0·013) and decreased appetite (1·42, 1·11–1·82; p=0·0058) were reported as symptoms, and longer in patients presenting with indigestion (0·71, 0·56–0·89; p=0·0033), back pain (0·77, 0·59–0·99; p=0·040), diabetes (0·71, 0·52–0·97; p=0·029), and self-reported anxiety or depression, or both (0·67, 0·49–0·91; p=0·011). Health system intervals were likewise longer with indigestion (0·74, 0·58–0·95; p=0·0018), back pain (0·76, 0·58–0·99; p=0·044), diabetes (0·63, 0·45–0·89; p=0·0082), and self-reported anxiety or depression, or both (0·63, 0·46–0·88; p=0·0064), but were shorter with male sex (1·41, 1·1–1·81; p=0·0072) and decreased appetite (1·56, 1·19–2·06; p=0·0015). Weight loss was associated with longer patient intervals (HR 0·69, 95% CI 0·54–0·89; p=0·0047).

**Interpretation:**

Although we identified no initial symptoms that differentiated people diagnosed with pancreatic cancer from those without pancreatic cancer, key additional symptoms might signal the disease. Health-care professionals should be vigilant to the possibility of pancreatic cancer in patients with evolving gastrointestinal and systemic symptoms, particularly in those with diabetes or mental health comorbidities.

**Funding:**

National Institute for Health Research and Pancreatic Cancer Action.

## Introduction

Pancreatic cancer is the tenth most common cancer in the UK, accounting for 3% of all new cancers in 2012.^1^ Incidence of the disease is rising, reflecting both an ageing population and an increased prevalence of obesity and diabetes.^2^ No effective screening methods are yet available, and most cases of pancreatic cancer have already invaded local structures or metastasised by the time patients are diagnosed.^3^ Survival from pancreatic cancer is very poor, with less than 5% of patients alive at 1 year.^1^ Despite advances in treatment and outcomes, a 10-year survival of 1% has remained unchanged for more than 40 years.^1^

The diagnostic pathway for pancreatic cancer comprises a series of stages or intervals that each contribute to the overall period of time between onset of symptoms and initiation of treatment.^4^ The total diagnostic interval comprises the patient interval (from onset of first symptom to first health-care consultation) and the health system interval (from first consultation via referral and investigations to diagnosis and initiation of treatment);^4^ shorter patient and health system intervals are associated with longer survival.^5^ In the past few years, reports have suggested that both intervals are longer for pancreatic cancer than for other common cancers, such as lung or urological cancer.^6^, ^7^, ^8^, ^9^

**Research in context**

**Evidence before this study**

We searched PubMed with the MeSH terms “symptom”, “diagnosis”, “pancreatic cancer”, and “interval” for all reports published before Dec 31, 2015. Little evidence exists for symptomatic presentations preceding a pancreatic cancer diagnosis: a US population-based case-control study identified common gastrointestinal symptoms associated with pancreatic cancer, and a qualitative study from the UK showed that symptoms of an intermittent nature might precede a pancreatic cancer diagnosis. No studies could be directly compared with our research because none reported factors associated with pancreatic cancer diagnosis and diagnostic intervals.

**Added value of this study**

We identified no first symptoms that aid identification of pancreatic cancer in people referred to hospital for suspicion of pancreatic cancer. However, as symptoms evolve, some subsequent symptoms can predict the diagnosis, such as jaundice, fatigue, change in bowel habit, weight loss, decreased appetite, and feeling different. Time to diagnosis was shorter in people with jaundice and decreased appetite as first symptoms, but was longer in people with comorbidities such as diabetes and common mental health problems.

**Implications of all the available evidence**

Our findings suggest that only people presenting with jaundice and decreased appetite as an initial symptom are investigated promptly. Primary care professionals could consider concurrent rather than sequential investigation, with CT and endoscopic ultrasound evaluation, of older people who present with indigestion that is atypical or associated with systemic symptoms. Health-care professionals could also have an increased awareness of the risk of pancreatic cancer in people with diabetes, and beware of misattributing potential symptoms of pancreatic cancer in those with mental health problems. The absence of strong symptom signals means that other strategies for early diagnosis, including the development of diagnostic biomarkers and improved access to imaging, are important.

Patients with pancreatic cancer can be symptomatic for many months before presentation.^10^ Not all individuals interpret their initial symptoms as serious, and some attribute them to ageing, lifestyle, or other comorbidities (Mills K, unpublished).^11^ International comparisons suggest perceived barriers to symptomatic presentation were highest in the UK;^12^ other potential influences include negative beliefs about cancer outcomes,^13^ and poor awareness of the risk of cancer.^14^, ^15^

The pathway to diagnosis in primary care can be complex, with delays arising when presentation is complicated by comorbidities, when referral is delayed or declined, or from false-negative investigation.^16^ Symptoms of possible pancreatic cancer much more commonly arise from benign or self-limiting disorders, and general practitioners (GPs) face a considerable challenge to assess patients whilst making efficient use of hospital-based resources. Much of the evidence about the predictive value of symptoms for cancer or their association with later diagnosis is from retrospective studies, or from general practice datasets,^17^, ^18^, ^19^ which are limited by quality of data recording. Little is known about the diagnostic pathways of people with pancreatic cancer or symptoms that are associated with less timely diagnosis. We therefore recruited a prospective cohort of patients with symptoms suggestive of pancreatic cancer near to the point of their referral, and investigated the symptoms and other clinical and sociodemographic factors associated with pancreatic cancer diagnosis and diagnostic intervals. We postulated that the diagnostic intervals would vary by symptoms and other patient factors.

## Methods

### Study design and patients

The SYMPTOM pancreatic study was done alongside the SYMPTOM lung^20^ and colorectal^21^ studies. We recruited patients from seven hospitals in the East (n=6) and North East (n=1) of England.

Potential participants were identified when referred to hospital via routine or urgent routes to a gastroenterology or hepatopancreatobiliary clinic, or relevant ultrasound departments. Referral letters were reviewed by a research nurse to identify patients aged 40 years or older whose referral mentioned one or more symptoms from a prespecified list of symptoms associated with pancreatic cancer (appendix). These patients were sent an information sheet, the questionnaire, and a reply-paid envelope. Patients were ineligible if they were already undergoing treatment for any cancer or had serious mental or physical disease deemed unsuitable for questionnaire completion. No reminder letters were sent to non-responders. A subsample of patients were recruited for a qualitative interview study; these results will be reported separately.

We obtained National Health Service (NHS) ethics approval (10/H0306/50) and clinical governance approval. All patients provided written informed consent with returned questionnaires.

### Data collection

The SYMPTOM pancreatic questionnaire asked for the initial symptom noticed by the participant, and then enquired about specific symptoms relevant to pancreatic cancer as an initial or subsequent symptom (appendix). The remaining sections enquired about other symptoms and patient factors, including demographic characteristics and comorbidities.

Participants' GPs completed a short proforma from the patient's clinical record, including dates of the first presentation with any of the ten specific symptoms within the previous 2 years, and the duration if recorded. We extracted data from hospital medical records for date and type of referral (urgent, routine, emergency, other); date of first specialist consultation; dates of investigations and their findings; date of diagnosis (histological, clinical); and dates of all multidisciplinary team meetings and their clinical decisions. Data extraction took place a minimum of 6 months after recruitment to ensure completion of investigation and initiation of treatment. We undertook double data extraction of a 5% sample of selected hospital data (dates of referral, first appointment, diagnosis, stage) and confirmed an acceptable level of agreement (>80% agreement for dates; >90% for diagnosis and stage).

We based our data collection, analysis, and reporting on the Aarhus statement for the conduct of cancer diagnostic studies,^4^ and STROBE guidelines for reporting observational studies.^22^

### Procedures

When available we used the date on the first confirmatory histology report as the date of diagnosis^4^ for both cancer (International Classification of Diseases codes 9–10) and non-malignant disorders, otherwise we used the date of diagnosis from the hospital medical record, including the outcomes of multidisciplinary team meetings. We categorised pancreatic cancer staging by TNM status at diagnosis when possible.^23^ When more than one staging report was available, we used the preoperative staging, which informed the initial treatment decision. Stage data were categorised into resectable or locally advanced and metastatic.^24^, ^25^ Complex diagnoses or cases with incomplete data were agreed by an adjudication group of clinicians (BB, FMW, JDE, GPR).

Demographic details comprised sex; age (as a continuous variable); ethnic origin (white *vs* non-white); smoking status; educational status; occupational status; living alone; and postcode, which we used to assign groups to quintiles of the Index of Multiple Deprivation (from quintile 1 [least deprived] to quintile 5 [most deprived]).^26^ Clinical variables relating to comorbidities comprised gastrointestinal disease; respiratory disease; self-reported anxiety or depression, or both; heart disease; diabetes; and arthritis. Family history of cancer was also identified (present *vs* absent).

All symptoms reported up to 2 years before diagnosis were included in the analysis.^27^ When provided, estimated dates were converted to exact dates with an adaptation of the C-SIM trial algorithm.^28^ We used exact dates when available and estimated dates otherwise. If a participant's unprompted initial symptom matched their response to a question about specific symptoms, they were given the corresponding symptom code and assigned the earliest of the dates provided. A first symptom and date of onset was thus identified for each participant. Many participants reported more than one initial symptom, termed multiple initial symptoms. Subsequent symptoms were defined as any symptom occurring after an initial symptom, but before diagnosis.

We defined the total diagnostic interval, or time to diagnosis, as the time from onset of the first symptom to date of diagnosis. When the date of first presentation to health care was known, we also calculated the patient interval, defined as the time from first symptom to first presentation, and the health system interval, defined as the time from first presentation to diagnosis.

### Statistical analysis

343 new cases of pancreatic cancer were diagnosed in Anglia and North Tees in 2005. Our original power calculation was based on recruitment of 200 participants with pancreatic cancer and 800 participants without cancer to provide more than 80% power to detect an 11% absolute difference in symptom prevalence (ie, 50% in cancer cases and 39% in non-cancer cases).

Descriptive analyses were done for the group as a whole, and by diagnostic group for patients with pancreatic cancer, other intra-abdominal or upper gastrointestinal tract cancers (herein referred to as other cancer), and no cancer. The main analyses focused on comparisons between pancreatic cancer and no cancer, with secondary analyses of all cancers (ie, pancreatic cancer plus other cancer) versus no cancer. Differences in demographic and clinical characteristics between diagnostic groups were assessed with Fisher's exact test and Wilcoxon rank-sum tests, as appropriate. Similarly, diagnostic intervals were compared between groups with a Wilcoxon rank-sum test. Clinically relevant demographics, comorbidities, symptoms present in the 2 years before diagnosis, and family history of cancer variables were a priori included in multivariable analyses to identify predictors of time to diagnosis. We excluded variables present in fewer than ten participants. We chose to analyse pancreatic cancer, other cancer, and no cancer groups collectively for all outcomes because at the time of presentation and referral the final diagnosis was unknown. Cox models were used to model the diagnostic intervals. Age was included as a linear effect; we confirmed that this was a reasonable assumption by adding a quadratic term to the model. Zero diagnostic intervals resulted from approximate dates and were included in the main analysis as half a day. We did analyses with Stata (version 13).

### Role of the funding source

The funders of the study had no role in the study design, data collection, data analysis, data interpretation, or writing of the report. The corresponding author had full access to all the data in the study and had final responsibility for the decision to submit for publication.

## Results

Between Jan 1, 2011, and Dec 31, 2014, 1543 people were approached, of whom 375 (24%) responded. 32 participants were reassigned to the pancreatic cohort from the SYMPTOM colorectal cohort, ten participants did not meet the eligibility criteria, and six participants had insufficient data for analysis, leaving a final cohort of 391 participants. The demographics of responders were similar to those of non-responders (responders: 52% male [n=194], median age 69 years [IQR 62–77]; non-responders: 49% male [n=413], median age 69 years [58–78]; appendix). Recruitment was more challenging than anticipated in our original power calculation, and the final total of cancer cases and non-cancer cases (excluding other cancers) provided more than 80% power to detect a 16% absolute difference.

119 (30%) participants were diagnosed with pancreatic cancer, 47 (12%) with other cancers, and 225 (58%) with no cancer (table 1). 16 (4%) patients were diagnosed with an intraductal papillary mucinous neoplasm; although these lesions are managed as premalignant disorders, we excluded these patients from the cancer group. 112 (94%) pancreatic cancers were confirmed by histology. 41 (34%) patients with pancreatic cancer had metastatic disease and 66 (56%) patients had non-metastatic disease; 12 (10%) patients had unstaged disease. The stage distribution for patients in our cohort who had pancreatic cancer was similar to the distribution for patients in the US National Cancer Database, in which 55·2% of patients had metastatic disease.^29^ No similar data exist for England. Of participants with stage data, 82 (69%) patients were staged preoperatively with an intention to treat, whereas 25 (21%) had been staged postoperatively.

**Table 1:** Diagnostic characteristics of participants (N=391)

		**n (%)**
Pancreatic cancer		119 (30%)
	Adenocarcinoma	98 (82%)
	Pancreatic neuroendocrine tumour	9 (8%)
	Ampullary carcinoma	8 (7%)
	Adenosquamous carcinoma	3 (3%)
	Mucinous carcinoma	1 (<1%)
Other cancer		47 (12%)
	Oesophageal adenocarcinoma	12 (26%)
	Cholangiocarcinoma and gallbladder carcinoma	11 (23%)
	Duodenal adenocarcinoma	4 (9%)
	Lymphoma	7 (15%)
	Hepatic adenocarcinoma	4 (9%)
	Metastases	3 (6%)
	Colorectal adenocarcinoma	2 (4%)
	Other*	4 (9%)
Non-cancer†		225 (58%)
	Nil abnormal	35 (16%)
	Hiatus hernia	28 (12%)
	Gallstones	25 (11%)
	Gastritis, duodenitis, ulcer	22 (10%)
	Oesophagitis, Barrett's oesophagus	20 (9%)
	Upper gastrointestinal polyp	18 (8%)
	Intraductal papillary mucinous neoplasm	16 (7%)
	Hepatitis (viral and other causes)	11 (5%)
	Liver disease (non-alcoholic)	10 (4%)
	Cholecystitis	9 (4%)
	Drug reaction	9 (4%)
	Pancreatitis	9 (4%)
	Pancreatic cyst (serous cystadenoma, pseudocyst)	8 (4%)
	Diverticular disease	6 (3%)
	Gastro-oesophageal reflux disease, clinical	4 (2%)
	Alcoholic liver disease	2 (<1%)
	Other	16 (7%)

* Breast, sarcoma, cancer of unknown primary, leukaemia (n=1 each).

† Percentages total more than 100% because patients might have had more than one diagnosis.

The diagnostic groups were similar in terms of sex, age, deprivation, education, ethnic origin, region, and smoking status (table 2). Compared with patients diagnosed with no cancer, those with pancreatic cancer were more likely to have diabetes and less likely to have arthritis (table 2).

**Table 2:** Sociodemographic characteristics

			**Pancreatic cancer (n=119)**	**Other cancer (n=47)**	**No cancer (n=225)**	**p value for pancreatic *vs* no cancer**
Sex						0·73
		Male	57 (48%)	25 (53%)	113 (50%)	
		Female	62 (52%)	22 (47%)	112 (50%)	
Age (years)			68 (42–93)	70 (47–89)	69 (40–89)	0·93
Highest education level						0·56
		Degree or diploma	45 (38%)	19 (40%)	81 (36%)	
		A Level, GCSE, O Level	42 (35%)	14 (30%)	71 (32%)	
		Other, none, or missing	32 (27%)	14 (30%)	73 (32%)	
Ethnic origin						0·30
		White	119 (100%)	44 (94%)	221 (98%)	
		Other or missing	0	3 (6%)	4 (2%)	
Smoking status						0·20
		Current	7 (6%)	5 (11%)	25 (11%)	
		Ex-smoker	52 (44%)	23 (49%)	83 (37%)	
		Never or missing	60 (50%)	19 (40%)	117 (52%)	
Deprivation (IMD quintile)						0·95
		Least deprived	37 (31%)	17 (36%)	71 (32%)	
		2nd quintile	41 (34%)	15 (32%)	77 (34%)	
		3rd quintile	23 (19%)	8 (17%)	40 (18%)	
		4th quintile	13 (11%)	4 (9%)	26 (12%)	
		Most deprived	3 (3%)	3 (6%)	10 (4%)	
		Missing	2 (2%)	0	1 (<1%)	
Comorbidities						
		Respiratory diseases*	17 (14%)	9 (19%)	38 (17%)	0·64
		Heart disease	7 (6%)	9 (19%)	26 (12%)	0·12
		Self-reported anxiety or depression, or both	14 (12%)	5 (11%)	39 (17%)	0·21
		Gastrointestinal diseases†	23 (19%)	2 (4%)	35 (16%)	0·37
		Diabetes	25 (21%)	4 (9%)	24 (11%)	0·014
		Arthritis	21 (18%)	9 (19%)	62 (28%)	0·047
		Any of the above	73 (61%)	24 (51%)	126 (56%)	0·36
Perceived family history risk						
		Diabetes	14 (12%)	6 (13%)	37 (16%)	0·27
		Cancer	38 (32%)	16 (34%)	66 (29%)	0·62
		Heart disease	20 (17%)	6 (13%)	63 (28%)	0·024
Referral route‡						
	General practitioner referral					
		Fast track	61 (51%)	26 (55%)	138 (61%)	
		Routine	2 (2%)	2 (4%)	50 (22%)	
		Unspecified or missing	19 (16%)	3 (6%)	9 (4%)	
		Hospital admission	2 (2%)	1 (2%)	1 (<1%)	
	Hospital referral					
		Accident and emergency	14 (12%)	11 (23%)	14 (6%)	
		Tertiary	13 (11%)	2 (4%)	5 (2%)	
		Internal	8 (7%)	2 (4%)	7 (3%)	
		Bowel screening	0	0	1 (<1%)	
	Region					0·59
		Cambridge	115 (97%)	43 (91%)	214 (95%)	
		North East	4 (3%)	4 (9%)	11 (5%)	

Values are n (%) or median (IQR), unless otherwise specified. A Level=Advanced Level. O Level=Ordinary Level. GCSE=General Certificate of Secondary Education. IMD=Index of Multiple Deprivation.

* Asthma, chronic obstructive pulmonary disease, others.

† Inflammatory bowel disease, peptic ulcer, irritable bowel syndrome.

‡ The p value for referral route is omitted because of incomplete information and some categories having very low frequencies.

Among the total cohort, 161 (41%) patients had a solitary initial symptom. 212 (54%) patients had multiple initial symptoms: 76 (19%) patients had two symptoms, 63 (16%) patients had three symptoms, and 73 (19%) patients had four or more symptoms; 18 (5%) patients reported no initial symptoms. Across the total cohort the most common symptoms were indigestion (n=224 [57%]), loss of appetite (n=207 [53%]), and fatigue (n=203 [52%]), but these were reported as first symptoms in only 136 (35%), 115 (29%), and 105 (27%) participants, respectively (table 3). Weight loss (n=175 [45%]), feeling different (n=174 [45%]), and change in bowel habit (n=170 [43%]) were also common symptoms, whereas jaundice (n=120 [31%]) and back pain (n=97 [25%]) were less commonly reported, and only reported as initial symptoms by 35 (9%) and 44 (11%) participants, respectively (table 3). Although not specifically enquired about in the questionnaire, a sizeable minority of patients reported a change in urine or stool colour, vomiting, unilateral torso pain, and bloating (table 3).

**Table 3:** Initial symptoms

	**Pancreatic cancer (n=119)**	All cancer*(n=166)	**No cancer (n=225)**	**p value for pancreatic *vs* no cancer**	**p value for all cancer *vs* no cancer**
**Symptoms in questionnaire**					
Indigestion	32 (27%)	48 (29%)	88 (39%)	0·024	0·036
Decreased appetite	33 (28%)	46 (28%)	69 (31%)	0·57	0·53
Fatigue	24 (20%)	35 (21%)	70 (31%)	0·030	0·027
Feeling different	25 (21%)	40 (24%)	62 (28%)	0·18	0·44
Change in bowel habits	32 (27%)	45 (27%)	50 (22%)	0·33	0·27
Weight loss	19 (16%)	25 (15%)	41 (18%)	0·60	0·41
Back pain	18 (15%)	22 (13%)	22 (10%)	0·14	0·28
Jaundice	14 (12%)	21 (13%)	14 (6%)	0·074	0·028
**Other symptoms reported by participants**					
Nausea or vomiting	4 (3%)	8 (5%)	20 (9%)	0·056	0·12
Change in urine or stool colour	13 (11%)	13 (8%)	11 (5%)	0·037	0·23
Unilateral torso pain	6 (5%)	7 (4%)	13 (6%)	0·78	0·49
Other pain	3 (3%)	5 (3%)	11 (5%)	0·29	0·35
Bloating	3 (3%)	4 (2%)	11 (5%)	0·29	0·21
Acute gastrointestinal illness	3 (3%)	3 (2%)	10 (4%)	0·37	0·15

Data are n (%), unless otherwise specified. Table shows symptoms present in ten or more participants, sorted by frequency in total cohort. Column percentages might total more than 100% because of multiple symptoms.

* Pancreatic and other cancers.

In this referred population, no initial symptoms were more frequently reported in the pancreatic cancer group than in the no cancer group; however, jaundice was more frequently reported in the all cancer group than in the no cancer group (table 3). Jaundice, change in urine or stool colour, fatigue, change in bowel habit, weight loss, decreased appetite, and feeling different were more frequently reported as subsequent symptoms in people diagnosed with pancreatic cancer than in those with no cancer (table 4).

**Table 4:** Subsequent symptoms

	**Pancreatic cancer**	All cancer*	**No cancer**	**p value for pancreatic *vs* no cancer**	**p value for all cancer *vs* no cancer**
**Symptoms in questionnaire**					
Indigestion	29/87 (33%)	43/118 (36%)	45/137 (33%)	1	0·60
Decreased appetite	41/86 (48%)	51/120 (43%)	41/156 (26%)	0·0011	0·0067
Fatigue	48/95 (51%)	58/131 (44%)	40/155 (26%)	0·0001	0·0012
Feeling different	32/94 (34%)	36/126 (29%)	36/163 (22%)	0·041	0·22
Change in bowel habits	36/87 (41%)	47/121 (39%)	28/175 (16%)	<0·0001	<0·0001
Weight loss	55/100 (55%)	68/141 (48%)	41/184 (22%)	<0·0001	<0·0001
Back pain	17/101 (17%)	25/144 (17%)	28/203 (14%)	0·50	0·37
Jaundice	51/105 (49%)	60/145 (41%)	25/211 (12%)	<0·0001	<0·0001
**Other symptoms reported by participants**					
Nausea or vomiting	3/115 (3%)	5/158 (3%)	7/205 (3%)	NA	1
Change in urine or stool colour	10/106 (9%)	12/153 (8%)	4/219 (2%)	0·0032	0·0081

Data are n/N (%), unless otherwise specified. Table shows symptoms present in ten or more participants, sorted by frequency in total cohort. The denominator (N) excludes patients that had the symptom as a first symptom. NA=not applicable.

* Pancreatic and other cancers.

A total diagnostic interval could be calculated for 373 participants (table 5). For patients with non-metastatic pancreatic cancer, the median total diagnostic interval for any first symptom was 108 days (IQR 47–222) compared with 136 days (86–323) for patients with metastatic disease (p=0·13). There were no differences in the total diagnostic interval, patient interval, or health system interval between people with pancreatic cancer and those with no cancer (table 5).

**Table 5:** Total diagnostic interval, patient interval, and health system interval for any symptoms

	**Total diagnostic interval (days)**			**Patient interval (days)**			**Health system interval (days)**		
	Median (IQR)	n	p value *vs* no cancer	Median (IQR)	n	p value *vs* no cancer	Median (IQR)	n	p value *vs* no cancer
**By diagnostic group**									
Pancreatic cancer	117 (57–234)	117	0·32	18 (0–37)	106	0·22	76 (28–161)	106	0·68
All cancer	112 (56–245)	164	0·19	11 (0–37)	150	0·078	75 (29–169)	150	0·74
No cancer	131 (66–284)	209	··	15 (1–62)	190	··	79 (30–156)	190	··
Total cohort	125 (66–268)	373	··	14 (0–55)	340	··	77 (29–160)	340	··
**By symptom**									
Jaundice	85 (44–161)	120	··	11 (2–33)	114	··	49 (23–95)	114	··
Change in bowel habits	113 (56–238)	170	··	14 (2–54)	159	··	70 (27–148)	159	··
Decreased appetite	114 (58–249)	207	··	13 (1–46)	190	··	75 (28–138)	190	··
Feeling different	124 (66–293)	174	··	12 (0–47)	163	··	83 (37–198)	163	··
Weight loss	125 (70–238)	175	··	22 (3–65)	160	··	71 (34–141)	160	··
Fatigue	131 (73–274)	203	··	15 (0–59)	181	··	77 (35–190)	181	··
Indigestion	138 (85–308)	224	··	18 (0–61)	210	··	88 (37–196)	210	··
Back pain	183 (99–325)	97	··	16 (0–76)	90	··	110 (49–264)	90	··

Total diagnostic interval, patient interval, and health system interval for any symptoms

Jaundice and decreased appetite were associated with a shorter total diagnostic interval, whereas indigestion, back pain, diabetes, and self-reported anxiety or depression, or both, were associated with a longer total diagnostic interval (table 6). Weight loss was associated with a longer patient interval (table 6). Male sex and decreased appetite were associated with a shorter health system interval, whereas indigestion, back pain, feeling different, diabetes, and self-reported anxiety or depression, or both, were associated with a longer health system interval (table 6).

**Table 6:** Predictors of total diagnostic interval, patient interval, and health service interval

		**Total diagnostic interval (n=370)**		**Patient interval (n=337)**		**Health system interval (n=337)**	
		HR (95 CI)	p value	HR (95 CI)	p value	HR (95 CI)	p value
Any symptom		··	··	··	··	··	··
	Indigestion	0·71 (0·56–0·89)	0·0033	0·79 (0·62–1)	0·051	0·74 (0·58–0·95)	0·018
	Decreased appetite	1·42 (1·11–1·82)	0·0058	1·15 (0·88–1·49)	0·31	1·56 (1·19–2·06)	0·0015
	Fatigue	1 (0·78–1·27)	0·97	0·88 (0·68–1·15)	0·35	1·03 (0·8–1·33)	0·83
	Weight loss	0·84 (0·66–1·06)	0·15	0·69 (0·54–0·89)	0·0047	0·9 (0·7–1·17)	0·44
	Feeling different	0·93 (0·74–1·17)	0·52	1·24 (0·96–1·6)	0·096	0·75 (0·58–0·96)	0·024
	Change in bowel habits	1·15 (0·91–1·45)	0·24	0·99 (0·78–1·26)	0·95	1·12 (0·87–1·44)	0·37
	Jaundice	1·38 (1·07–1·78)	0·013	1·29 (0·99–1·68)	0·056	1·19 (0·91–1·55)	0·20
	Back pain	0·77 (0·59–0·99)	0·040	0·85 (0·65–1·12)	0·25	0·76 (0·58–0·99)	0·044
Sex (ref=female)		··	··	··	··	··	··
	Male	1·23 (0·98–1·56)	0·080	0·93 (0·73–1·18)	0·55	1·41 (1·1–1·81)	0·0072
Age at diagnosis (10 years)		1·1 (0·98–1·22)	0·10	1·08 (0·97–1·22)	0·16	1 (0·9–1·12)	0·95
IMD quintile (ref=least deprived)		··	0·51	··	0·42	··	0·35
	2nd quintile	0·94 (0·73–1·22)	··	0·87 (0·66–1·16)	··	0·84 (0·63–1·1)	··
	3rd quintile	1·17 (0·85–1·6)	··	1·1 (0·79–1·53)	··	1·08 (0·77–1·5)	··
	4th quintile	1·23 (0·84–1·82)	··	1·26 (0·84–1·9)	··	0·97 (0·64–1·45)	··
	Most deprived	0·91 (0·52–1·6)	··	1·16 (0·66–2·06)	··	0·68 (0·39–1·19)	··
Diabetes		0·71 (0·52–0·97)	0·029	1·06 (0·76–1·49)	0·72	0·63 (0·45–0·89)	0·0082
Gastrointestinal comorbidity		1 (0·74–1·37)	0·99	0·97 (0·69–1·35)	0·84	1·22 (0·88–1·69)	0·23
Self-reported anxiety or depression, or both		0·67 (0·49–0·91)	0·011	1·01 (0·73–1·41)	0·95	0·63 (0·46–0·88)	0·0064
Family history of cancer		0·81 (0·64–1·03)	0·083	0·93 (0·73–1·2)	0·59	0·79 (0·61–1·02)	0·067

Proportional hazards Cox model adjusted for all variables in the table plus smoking status (never *vs* current *vs* ex-smoker) and living alone (yes *vs* no). The model includes patients with all diagnoses. HR=hazard ratio. IMD=Index of Multiple Deprivation.

## Discussion

To our knowledge, this is the first large prospective cohort study to investigate factors associated with diagnostic intervals for pancreatic cancer and compare them with people with similar symptoms found not to have cancer. No initial symptoms were associated with pancreatic cancer in this referred population, but a wide range of subsequent symptoms were associated with pancreatic cancer. Jaundice and appetite change were both associated with shorter total diagnostic intervals and health system intervals, suggesting that they are recognised more promptly by health-care professionals and investigated more quickly than other symptoms. By contrast, indigestion and feeling different had longer health system intervals, suggesting they might be managed in other ways, including alternative diagnostic pathways or empirical treatments. Back pain, diabetes, and self-reported anxiety or depression, or both, were all associated with longer health system intervals, suggesting that health-care professionals might misattribute symptoms in these groups to other causes. Weight loss was the only symptom associated with a longer patient interval, perhaps showing that patients make alternative explanations for this symptom and delay seeking help.

Key strengths of this study were the prospective design, collection of data from several sources (patient reports, and primary care and specialist records), and compliance with recommendations in the Aarhus statement^4^ and STROBE guidelines.^22^ Because we were analysing patient-reported symptoms, we defined the date of onset of initial symptoms with patient-reported rather than primary care-reported date. Ideally, we would have recruited patients from primary care before referral; however, this approach would have major logistical and resource implications in the identification of a large prospective cohort of patients with symptoms of possible pancreatic cancer in order to capture a sufficient number of patients with cancer. Instead, we recruited patients as early as possible in the hospital setting. This method had the added benefit of allowing us to recruit patients referred from other specialists, and those presenting as emergencies, although we might have under-recruited this group when compared with routes to diagnosis data for 2013.^30^ We recruited patients with a broad range of socioeconomic, educational, and occupational levels, although our cohort had a slightly lower proportion of more deprived patients than in the national population. Our recruitment process meant that some patients transpired to have other upper gastrointestinal cancers and 16 people had intraductal papillary mucinous neoplasms.^31^ We do not consider this aspect a weakness, rather, it serves to highlight the complexity and challenges in the diagnosis of pancreatic cancer, and how similarly different gastrointestinal cancers can present.

The main limitation of our study is the response rate of 24%; however, this is similar to the response rate in other prospective studies of lung and colorectal cancer symptoms.^20^, ^21^ We contacted the target population during the course of their investigation and a possibility is that some people were not able to complete a questionnaire at this worrying time. We might have under-recruited people who presented as an emergency. However, the demographics of non-responders were similar to those of responders, and the proportion of patients with metastatic pancreatic cancer was similar to international data,^29^ suggesting that our cohort was reasonably representative. Although this was a large cohort for this difficult-to-diagnose disease, we had insufficient power to examine specific clusters of symptoms and their associations with outcomes. A much larger prospective study would be needed to gain sufficient power, although this would be logistically challenging to achieve.

A systematic review^5^ provided evidence of an association between shorter times to diagnosis and more favourable outcomes for some cancers, such as breast and colorectal cancer. For pancreatic cancer, two of the five identified studies reported a positive association, whereas three reported no association.^5^ Our study found that the median patient interval for the total cohort was 14 days and the median health system interval (combining primary and secondary care) was 77 days. Although we included both cancer and non-cancer cases, these findings are similar to those from the National Audit of Cancer Diagnosis in Primary Care,^9^ which showed a median patient interval of 19 days (IQR 1–61). The moderately long health system interval should be set in the context of high rates of multiple GP visits recorded in the National Patient Experience Survey for patients with pancreatic cancer.^32^ The only UK primary care database study showed that jaundice was the most predictive symptom for pancreatic cancer; only one other symptom, weight loss, when present with another non-jaundice symptom, increased the positive predictive value to more than 2%.^17^ In our study, weight loss was an uncommon first symptom but a common subsequent symptom; therefore, even participants with more than one initial symptom would not necessarily be considered to have an increased risk of pancreatic cancer. In our qualitative study (Mills K, unpublished) people described subtle changes in appetite and often attributed their symptoms to dietary choices or an acute illness, prompting changes in type and frequency of meals. Appetite loss was the only symptom associated with a shorter health system interval, suggesting that health-care professionals might also have been alert to the significance of these changes.

Our findings show that there are subtle differences in the effect of symptoms and patient factors on patient and health system intervals, with some clear implications for policy makers and clinicians. That no initial symptoms were predictive of pancreatic cancer emphasises the challenges of triaging referrals for suspected pancreatic cancer. Although the alarm symptom jaundice was more frequently reported by patients in the all cancer group than by those with no cancer, it was reported as the first symptom in less than 12% of cases, and reported much more frequently as a subsequent symptom (43% of cases). We did not identify any other strong individual symptom signals of pancreatic cancer, suggesting that awareness campaigns should include messages that emphasise the importance of multiple, often intermittent, upper gastrointestinal symptoms (eg, decreased appetite and indigestion) and systemic symptoms (eg, fatigue and weight loss), and the evolution of symptoms over time.^33^ Revised NICE guidelines^34^ for early detection of pancreatic cancer support this premise, and have also lowered the threshold for referral.

Our study also shows that only people presenting with jaundice and decreased appetite as an initial symptom receive a diagnosis more promptly than do those presenting with other symptoms. Primary care professionals could consider concurrent rather than sequential investigation, with CT and gastroscopy, of older people who present with indigestion that is atypical or associated with systemic symptoms.^35^ Primary care professionals could also have an increased awareness of the risk of pancreatic cancer in people diagnosed with diabetes, particularly those with normal weight without the typical features predisposing to insulin resistance. All health-care professionals should remain alert to possible pancreatic cancer in people with back pain, and beware of misattributing potential symptoms of pancreatic cancer in those with self-reported anxiety and depression, or both. The increasingly widespread use of clinical decision support in primary care can also be informed by our findings,^36^, ^37^ but further research is needed, alongside GPs and specialists, to identify mechanisms by which patients can be identified, referred, and diagnosed in the most timely and appropriate way. The absence of strong symptom signals means that other strategies for early diagnosis, including the development of diagnostic biomarkers and improved access to imaging, are important. Nevertheless, these data provide support for more targeted investigation of suspicious symptoms to identify pancreatic cancer at an earlier and more amenable stage.
