# Supplementary material for: Symptoms and patient factors associated with diagnostic intervals for pancreatic cancer (SYMPTOM pancreatic study): a prospective cohort study
Source: Lancet Gastroenterol Hepatol. 2016 Oct 4;1(4):298–306. doi: 10.1016/S2468-1253(16)30079-6 (PMC6358142; doi:10.1016/S2468-1253(16)30079-6)
Supplement: Supplementary appendix [file mmc1.pdf]

THE LANCET
Gastroenterology & Hepatology

Supplementary appendix

This appendix formed part of the original submission and has been peer reviewed. We post it as supplied by the authors.

Supplement to: Walter FM, Mills K, Mendonça SC, et al. Symptoms and patient factors associated with diagnostic intervals for pancreatic cancer (SYMPTOM pancreatic study): a prospective cohort study. *Lancet Gastroenterol Hepatol* 2016; published online Oct 4. http://dx.doi.org/10.1016/S2468-1253(16)30079-6.

Appendix A

Aged 40 and over, via:

(i) Emergency admissions and fast track jaundice service referrals

Include: ALL

(ii) Fast track/urgent clinic and routine referrals

Include:

- Abdominal pain
- Abdominal mass
- Jaundice
- Expressed concern about possibility of cancer
- Unexplained weight loss

Exclude:

- Review of established diagnosis where there is no cancer concern
- Previous GI cancer
- Known infective diarrhoea (e.g. after recent foreign travel)
- Any planned surgery unrelated to the pancreas i.e. cholecystectomy, hernia repair
- Stated suspicion of gallstones
- Dyspepsia as the presenting symptom,

Appendix B

The questionnaire starts with: '*We are interested in symptoms which you think are related to your recent referral to hospital. The following questions are about when you first noticed a symptom and when you first told your GP or nurse about it. Please give an exact date if you can. Otherwise please give your best estimate (for example, approximately how long ago, the month or the season). You may wish to refer to your diary or calendar if you have it with you'.* The first question was: 'What was the first thing or symptom you noticed that made you think something might be wrong?' Seven specific symptoms followed: jaundice (yellow colour to your skin); back pain; indigestion or heartburn or persistent tummy ache 'that wasn't normal for you' (categorised as 'indigestion'); plus decrease in appetite, unexplained weight loss, fatigue or tiredness 'that is unusual for you' (categorised as 'fatigue'), and feeling different 'in yourself' from usual.

Appendix Table 1: Comparison of age and gender demographics for responders and non-responders of the pancreatic cohort

Demographic Feature	Returned (n=375)	Not returned (n=1168)	Total (n=1543)	p-value
**Gender, n (%)**				0.32
Female	181 (48%)	438 (51%)	619 (50%)	
Male	194 (52%)	413 (49%)	607 (50%)	
N Missing		317	317	
**Age at identification**				0.50
Median, IQR	69 (62-77)	69 (58-78)	69 (59-78)	
N Missing		86	86	
